# Childhood obesity, overweight and underweight: a study in primary schools in Milan

**DOI:** 10.1007/s40519-013-0036-9

**Published:** 2013-05-04

**Authors:** Renata Bracale, Laura Milani, Emanuela Ferrara, Claudia Balzaretti, Alessandra Valerio, Vincenzo Russo, Enzo Nisoli, Michele O. Carruba

**Affiliations:** 1 Department of Medicine and Sciences for Health (Dip. M.S.S.), Molise University, Via Giovanni Paolo II, 86100 Campobasso, Italy; 2 Department of Medical Biotechnology and Translational Medicine, Center for Study and Research on Obesity, University of Milan, Milan, Italy; 3 Institute of Communication, Behaviour and Consumption, “Giampaolo Fabris”, IULM University, Milan, Italy; 4 Milano Ristorazione S.p.A, Milan, Italy; 5 Department of Veterinary for Health, Animal Production and Food Safety (VESPA), University of Milan, Milan, Italy; 6 Department of Molecular and Translational Medicine, Brescia University, Brescia, Italy; 7 Food Consumption Observatory, IULM University Foundation, Milan, Italy

**Keywords:** Obesity, Overweight, Underweight, Children, Primary schools, Immigrants, Physical activity, TV watching

## Abstract

**Aims:**

The study aims to assess the prevalence of obesity, overweight and underweight in children enrolled in government primary schools (6–11 years of age) in the city of Milan, Italy.

**Methods:**

One hundred and nine schools were randomly selected for the study. A cross-sectional study was conducted between March and June 2008. A survey was conducted using 16,588 questionnaires completed by parents. Anthropometric data (reported) of both parents and children and information on levels of physical activity and time children spent watching television (TV) were obtained.

**Results:**

In the total sample, parents are predominantly (75.0 %) of normal weight (M: 55.2 %; F: 79.1 %), 16.8 % are overweight (M: 36.9 %; F: 12.6 %), 4.0 % are obese (M: 6.6 %; F: 3.5 %) and 4.2 % are underweight (M: 1.3 %; F: 4.8 %). Among children, 68.7 % are of normal weight (M: 68.2 %; F: 69.2 %), 14.7 % are overweight (M: 15.3 %; F: 14.2 %), 4 % are obese (M: 4.5 %; F: 3.4 %), 11.8 % are underweight (M: 11.2 %; F: 12.5 %) and 0.8 % are severely thin (M: 0.9 %; F: 0.7 %). Children practice physical activity once or twice/week (48.3 %), three to four times/week (38.9 %) or five to seven times/week (8.9 %), while 3.9 % of children do not do any exercise. Most children (85.3 %) watch TV from 30 min to 2 h/day.

**Conclusion:**

Contrary to the reported national average, the study shows the presence of only moderate levels of above-average weight and obesity among children. However, it remains important to monitor this phenomenon to raise awareness and to design programs of prevention throughout the country.

## Introduction

The prevalence of overweight in children has increased in almost all countries for which data are available. Obesity and overweight have increased worldwide and even more dramatically in economically developed countries and in urbanized populations [1]. The World Health Organization (WHO) has described obesity as the worst non-infectious epidemic in history.

Obesity is a potentially serious disease because of its impact on the physical and psychological health of children; in addition, most obese children become obese adults. In fact, more than half of the obese 6-year-old children remain obese in adulthood, whereas only 10 % of children of the same age and normal weight become obese in adulthood [2]. Childhood overweight not only increases the risk of obesity in adulthood, but is also the leading cause of pediatric hypertension and is associated with type 2 diabetes mellitus [3].

Childhood overweight etiology includes modifiable and non-modifiable risk factors [4]. The modifiable risk factors include lack of regular exercise, high frequency of TV watching, computer usage or usage of other screen media, low parental educational level, non-working parents, over-consumption of high-calorie foods, snacking while watching TV or doing homework and over-exposure to advertisements of high-calorie foods [5, 6]. The common non-modifiable cause of obesity is genetic disposition, and, in particular, a greater risk of obesity exists among children whose parents are overweight or obese [7]. Other influential factors are changes in the living conditions of children and their families, urbanization and migration [8].

Interventions for reducing childhood obesity aimed at modifiable risk factors have been extensively indicated in the literature as key in reversing the detrimental health effects impacting the children’s life quality [9].

In Italy, several surveys conducted both at the local and regional levels have highlighted some geographical differences in pediatric obesity levels [10, 11]. At the national level, 24 % of 8- to 9-year-old children are overweight and 12 % are obese, according to IOTF criteria [12]. Above all, the comparison between regions is difficult, because of differences in methodology, in defining the cutoff points and the incomplete geographic coverage.

Considering the geographic differences reported in the emerging pediatric obesity levels in Italy, the aim of this study is to investigate the prevalence of obesity and overweight in a sample of children enrolled in government primary schools in the city of Milan, Italy, and to understand better the modifiable risk factors related to this prevalence, including sedentary lifestyle, demographic, and socio-cultural and ethnic factors.

## Subjects and methods

### Subjects

The survey was carried out in all primary schools using the service of “Milano Ristorazione” public catering service, which is a provider of lunchtime meals, in March 2008. The questionnaire was delivered to the students’ families after the authorization of the Italian Department Educational School, presentation of the project by a letter of information and upon approval of the Regional School of Lombardia.

A total of 45,000 questionnaires were consigned to children to be filled out by their parents. In this survey design, initial units were represented by 147 primary schools, but only 109 schools returned the questionnaires. The final units were families with school-age children (ages 6–11 years). A total of 23,275 questionnaires were returned, of which only 16,588 were correctly completed by parents and subsequently analyzed.

**Fig. 1:**
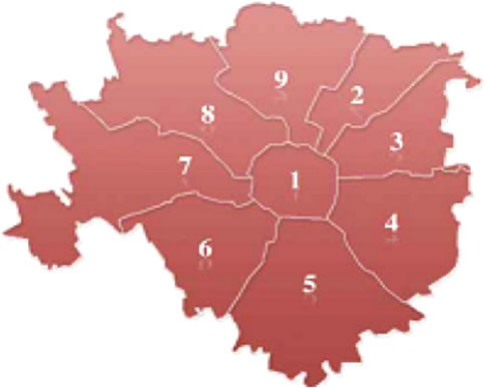
Zones of Milan, Source, www.comunedimilano.it

More than 1,300,000 Milanese inhabitants [13] are distributed over a territory divided into nine zones (Fig. 1). Where it was possible to identify the number and the incidence of foreign residents, 20.6 % of the families that responded to the survey represented foreign residents.

The study involved about 47 % of the total population of children in Milan enrolled in public primary schools during the 2007–2008 scholastic year. In relation to all the children in Milan, in both public and private schools, the report referred to 36 % of the target population.

### Survey method

The survey included the following information: parents’ and children’s data; the family structure and composition; the level of parental education and employment; the parents’ and children’s anthropometric measures; the frequency of the children’s physical activity; and the parents’ and children’s TV watching habits.

### Anthropometric measurements

Parents’ and children’s body mass index (BMI) was based on the reported height and weight and calculated as weight (kg)/height (m)^2^. Self-reported weight and height have been described as being acceptable measurements for the determination of BMI [14]. Categories for parental BMIs were based on the World Health Organization guidelines: underweight (<18.5), normal weight (18.5–24.9), overweight (25–29.9) and obese (≥30.0) [1].

With reference to children’s BMI, the cutoff points for ‘overweight’ and ‘obese’ were sourced from Cole et al. [15], and the cut-off points for ‘underweight’ were those for children and adolescents sourced from Cole et al. [16]. BMI was calculated using specific classifications based on sex and age.

### Statistics

All analyses were performed using SPSS package for Windows (v. 15). Frequency distributions and percentages were generated for all variables and used for descriptive purposes. To study the weights of each factor on the children’s BMI, the differences were analyzed by applying the Chi-square test. It was used to examine the relationships among children’s BMI and children’s weekly physical activity, parents’ educational level, children’s daily time spent watching TV and parents’ BMI, respectively. A value of *p* < 0.05 was used to denote significant differences.

## Results

Data were collected from 75 % of the schools using the services of “Milano Ristorazione” public catering service. The schools which showed adherence to the survey represented half of the primary school structures throughout the territory of Milan. Among respondents, there was a response rate of 50 %.

The sample comprised primarily of women (82.5 %), while men represented only 17.5 %.

Most of the parents were in the age group between 30 and 50 years (94.2 %). Parents with one child contributed 26 %; parents with two, 53.5 %; parents with three, 16.3 %; and parents with more than three sons, only 4.2 %. Larger families (three or more children) were mostly attributable to foreign residents (29.0 vs. 19.8 %). Most of the sample was married (81.6 %), 9 % reported being divorced or separated, while the remaining portion of the sample was unmarried. Most individuals in the sample were employed (79.7 %), while 2.2 % were unemployed, 0.2 % retired, 4.7 % otherwise occupied and 12.9 % represented housewives. The collected data on educational level reveal that 33.7 % of the sample earned a degree, 46.3 % had a high school diploma, 15.4 % had a middle school diploma and 1.4 % of respondents had no educational qualifications.

In the sample, 83.6 % were Italians and 15.1 % foreigners. Of those reported as foreigners, 32 % were from Asia, 22.4 % from Central and South America, 15.5 % from a European country, 17.8 % from Africa and only 0.1 % from Oceania or were stateless.

According to parents’ BMI, 75 % were of normal weight (M: 55.2 %; F: 79.1 %), 16.8 % were overweight (M: 36.9 %; F: 12.6 %), 4.0 % were obese (M: 6.6 %; F: 3.5 %) and 4.2 % were underweight (M: 1.3 %; F: 4.8 %) (data no show). Another difference existed between Italian parents’ BMI and foreign resident parents’ BMI. The latter showed a higher prevalence of overweight (25.8 %) and obesity (6.2 %) and a lower prevalence of normal weight 65.9 % (Table 1). It also seems relevant to report that among African people, 45.7 % were overweight and 10.8 % were obese. The survey reveals a stratification of the BMI classes in relation to parents’ age and to the geographic area of the families within the city. In Zone 1 (Fig. 1), which represents the historical center of Milan, parents with BMI <18.5 appear frequently. Areas with a higher prevalence of overweight and obesity are Zones 2, 6 and 9, which are also the areas with the highest incidence of foreign residents [13].

**Table 1:** Parents’ BMI according to their nationality and gender

	Italian						Foreign					
	Total		Male		Female		Total		Male		Female	
	*n*	%	*n*	%	*n*	%	*n*	%	*n*	%	*n*	%
Underweight	615	4.6	28	1.3	587	5.2	54	2.1	7	1.1	47	2.4
Normal	10359	76.8	1200	56.2	9159	80.7	1716	65.9	322	51.8	1394	70.4
Overweight	2030	15.0	770	36.0	1260	11.1	672	25.8	248	39.9	424	21.4
Obese	485	3.6	138	6.5	347	3.1	161	6.2	45	7.2	116	5.9
Total	13489	100.0	2136	100.0	11353	100.0	2603	100.0	622	100.0	1981	100.0

All parents have children (M: 50.3 %; F: 49.7 %) between ages 6 and 10 years. According to the data on height and weight reported by parents, the children’s BMI are as follow: 68.7 % are of normal weight (M: 68.2 %; F: 69.2 %), 14.7 % are overweight (M: 15.3 %; F: 14.2 %), 4 % are obese (M: 4.5 %; F: 3.4 %), 11.8 % are underweight (M: 11.2 %; F: 12.5 %) and 0.8 % are severely thin (M: 0.9 %; F: 0.7 %) (Table 2). A comparison of the BMI of the children of Italians with those of foreign residents shows an emergence of the same trend as that existing for parents: the frequency of obesity among foreign resident children is nearly three times that of Italian children (8.7 vs. 3.0 %). Even within normal-weight children, a notable difference was observed (foreign vs. Italian: 60.5 vs. 70.3 %). The highest rates of overweight and obese children were among families from Africa, Asia and both Central and South America (Table 3).

**Table 2:** Children’s BMI according to their gender

	Total		Male		Female	
	*n*	%	*n*	%	*n*	%
Severely thin	125	0.8	71	0.9	54	0.7
Underweight	1963	11.8	933	11.2	1030	12.5
Normal	11399	68.7	5687	68.2	5712	69.2
Overweight	2444	14.7	1275	15.3	1169	14.2
Obese	657	4.0	373	4.5	284	3.4
Total	16588	100.0	8339	100.0	8249	100.0

**Table 3:** Children’s BMI according to their nationality and gender

	Italian						Foreign					
	Total		Male		Female		Total		Male		Female	
	*n*	%	*n*	%	*n*	%	*n*	%	*n*	%	*n*	%
Severely thin	96	0.7	61	0.9	35	0.5	29	1.1	10	0.7	19	1.4
Underweight	1698	12.2	818	11.7	880	12.8	265	9.7	115	8.4	150	11.0
Normal	9750	70.3	4899	70.2	4851	70.4	1649	60.5	788	57.8	861	63.2
Overweight	1900	13.7	974	14.0	926	13.4	544	20.0	301	22.1	243	17.8
Obese	419	3.0	224	3.2	195	2.8	238	8.7	149	10.9	89	6.5
Total	13863	100.0	6976	100.0	6887	100.0	2725	100.0	1363	100.0	1362	100.0

A higher frequency of overweight and obesity was shown among children within Zones 2, 4, 6 and 9, and the lowest BMI was reported again within Zone 1.

**Fig. 2:**
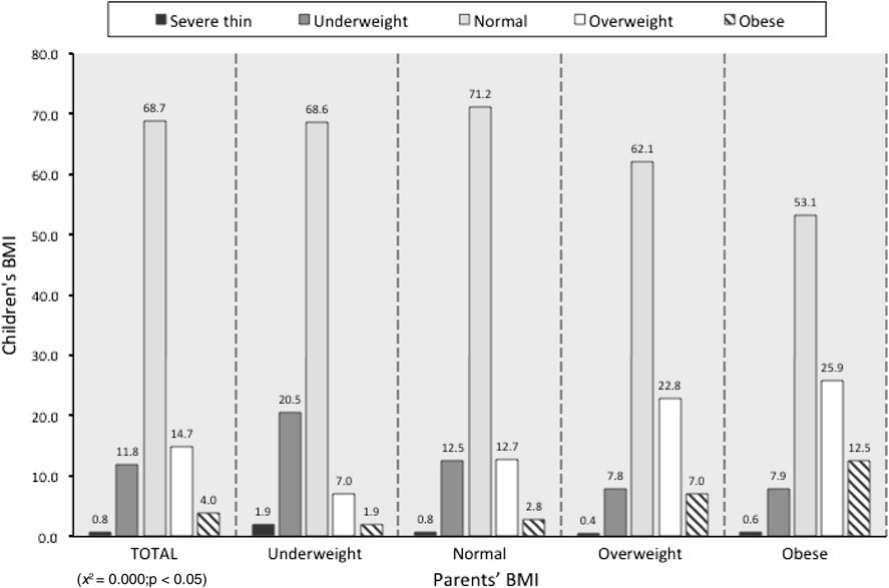
Children’s BMI versus parents’ BMI, *x* = parents’ BMI, *y* = children’s BMI. Data are expressed as percentages (*χ* ^2^ = 0.000; *p* < 0.05)

**Fig. 3:**
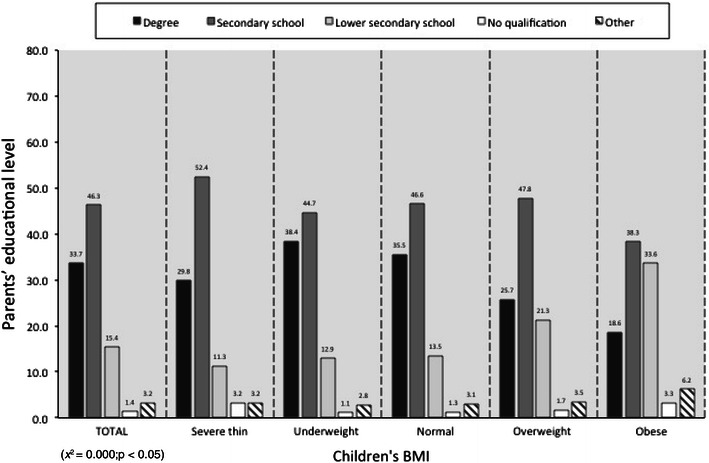
Parents’ educational level vs children’s BMI, *x* = children’s BMI, *y* = parents’ educational level. Data are expressed as percentages (*χ* ^2^ = 0.000; *p* < 0.05)

Children’s BMI was correlated to parents’ BMI (*p* < 0.05) (Fig. 2), while an inverse correlation (*p* < 0.05) between children’s BMI and parents’ educational level emerged (Fig. 3).

**Fig. 4:**
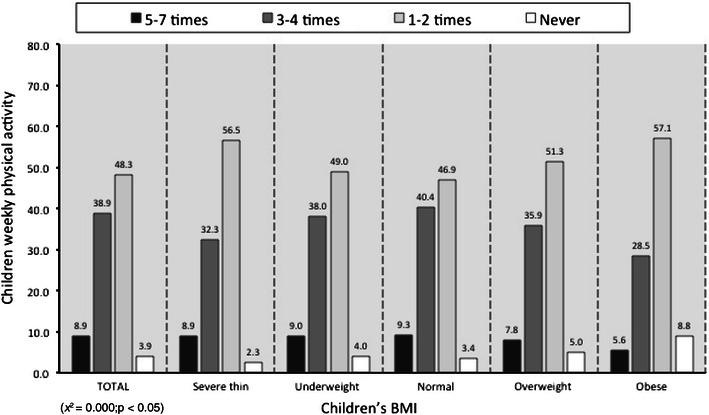
Children weekly physical activity vs children’s BMI, *x* = children’s BMI, *y* = children weekly physical activity. Data are expressed as percentages (*χ* ^2^ = 0.000; *p* < 0.05)

With respect to weekly physical activity, 48.3 % of children were physically active once or twice/week, 38.9 % three to four times/week and 8.9 % five to seven times/week, while 3.9 % did not do any exercise. Data analyses showed that a low frequency of physical activity for children corresponded with an increase in their BMI (*p* < 0.05) (Fig. 4).

**Fig. 5:**
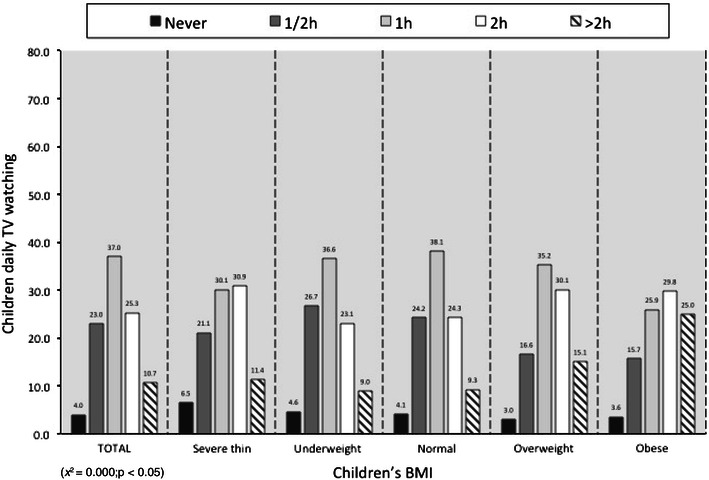
Children daily TV watching vs children’s BMI, *x* = children’s BMI, *y* = children daily TV watching. Data are expressed as percentages (*χ* ^2^ = 0.000; *p* < 0.05)

With regard to daily screen usage time of children during school days, parents stated that children spent half an hour (23.0 %), 1 h (37.0 %), 2 h (25.3 %) or more than 2 h (10.7 %) watching TV; a small percentage of children (4 %) never watched TV (Fig. 5). The children’s BMI correlated with greater hours of daily TV watching (*p* < 0.05).

The last consideration was the high prevalence of underweight (11.8 %) and severe thinness (0.8 %) among children in the city of Milan. These data were in contrast with that of the national average [17].

## Discussion

Childhood obesity in Italy is distributed differentially across the country (north, center and south), and the prevalence in the south is twice that in the north [17].

Several surveys, which were conducted both at the local and regional levels, have highlighted geographic differences in the obesity phenomenon [10, 17]. Data for some areas, including the Lombardia Region, in the north of the country, are not available. The present study has two main objectives: to evaluate the prevalence of childhood overweight and obesity in a sample of school children living in Milan and to examine socio-cultural, parental and lifestyle factors associated with children’s BMI that contribute to the increase in the risk of obesity.

Although our survey protocol was based on a different methodology compared to that used for studies at the national level [17], our data reflect findings reported in the existing literature allowing us to draw some specific conclusions about the Italian metropolitan area. In fact, some authors have proposed useful algorithms for analysis of the adult population, based on self-reported information on weight and height. In adults, bias in self-reported information may depend on demographical, cultural, social and health characteristics. Nyholm et al. [18] have concluded that self-reported BMI may be a useful instrument when adjusted for variables that are predictive for misreporting.

The data obtained in our survey reveal a better health situation than that of the Italian average with regard to both overweight and obesity levels. Almost all the parents seemed to be of normal weight (75.0 %), 16.8 % were overweight and 4.0 % were obese. Comparing this with the Italian average [19], which indicates that normal-weight adults are 51.5 % and overweight and obese adults are 35.5 and 9.9 %, respectively, these data present a situation of relatively better health for this sample. The same conclusion could be made considering the status of children in the study sample. Overweight and obese children were found to be 14.7 and 4.0 %, respectively, in contrast to the corresponding Italian national average of 24 and 12 %. This observed difference is especially notable when considering some south Italian regions, such as Campania, Molise and Sicilia, where childhood obesity reaches the highest levels (21, 14.8 and 12.8 %, respectively) [20]. Some authors recently attributed not having breakfast, sedentariness and TV watching as harmful behaviors connected to an increased risk of obesity [21]. Binkin et al. [12] have reported that these unhealthy lifestyles are especially present in the south Italian regions.

Several epidemiological and intervention studies have suggested that the time spent watching TV may be a relevant element to the increase of overweight and obesity among children and adolescents [22]. The time spent watching TV contributes to overweight or obesity through reducing physical activity and increasing calorie intake from eating high-fat and high-sugar foods [23]. From the survey reported here, it emerges that most of the children spent less time watching TV (from ½ to 2 h/day) compared to the national average, which showed that about 29 % of Italian children used screen media for 2–2.5 h/day, 25 % for 3–3.5 h/day and 23 % >4 h/day [17]. Only 3.3 % of children watched TV more than 4 h/day, representing the highest risk population for becoming obese. The small amount of time spent watching TV could be a protective factor for those children living in Milan.

The progressive reduction in daily physical activity is an established risk factor in developing childhood obesity [24]. Children who habitually practice physical activity (more than three to four times/week) are more frequently of normal weight (Fig. 4). The key causes of overweight and obesity incorporate not only individual behaviors regarding nutrition and physical activity, but also include environmental factors such as the residential area, the traffic situation and the national programs regarding nutritional and social aspects. With reference to our sample, it appears useful to highlight another plausible protective factor influencing the BMI deviation from the Italian average: all the children who took part in the survey usually have at least one balanced meal, for 6 days of a week, at the school canteen.

Additional influential factors, which appear to be relevant and are discussed among socio-medical and public health experts, include increased urbanization and motorization, changes in the environmental living of children and their families and being an immigrant with its specific biosocial and cultural implications especially among children of immigrants [8]. In our sample, there were high frequencies of overweight and obesity among foreign resident adults and their children (Tables 1, 3). Among foreign adult residents living in Milan, the African population was the most overweight and obese (45.7 and 10.8 %, respectively); also those coming from Oceania and Central and South America showed higher levels of above-average weight. The same relationship existed when comparing the children’s BMI and the country of origin. There was a high percentage of overweight and obese children from Africa, Asia and Central and South America. Our survey is consistent with the current literature, which shows that in economically developed countries, children belonging to specific ethnic groups are at greater risk for above-average weight or obesity [1].

According to some studies, low socioeconomic and minority status of migrant people seem to be associated with increasing chronic distress and high morbidity and mortality [25]. The difficult process of acclimation and acculturation may also contribute to make this population more vulnerable. Behavioral changes in culture and diet are associated with migration: the abandonment of a balanced traditional diet and the Westernization of dietary habits can lead immigrants to obesity and related diseases such as diabetes, hypertension and cardiovascular diseases [26, 27]. Data from other countries show that adolescent immigrants from Asia and Spain who were born in USA had twice the possibility of becoming obese than the first generation immigrants [28], and, in another study, immigrant children from Turkey and former Yugoslavia had a higher possibility of being overweight than did native Austrian children [25].

These results are notable with regard to the level of education of the parents in Milan. The number of parents with degree (33.7 %) is higher than that of the national average and only 1.4 % has no educational qualifications (Fig. 3). These data have two possible explanations. It is possible that parents with higher educational levels may be more sensitive and willing to respond to the questionnaire, or there may be a greater representation of people with degree in the city of Milan. The literature frequently shows the relationship between educational level and BMI, with a negative trend in countries with different socioeconomic context. High socio-cultural status correlates with high BMI in developing countries [29]. However, an inverse relationship exists between these two parameters in developed Western society: overweight and obesity are prevalent among subjects with a lower educational level.

Urban planning emerges as an additional factor influencing the risk of developing overweight and obesity [26]. In Milan, the highest levels of overweight and obesity among parents and children are present in the most peripheral areas of the city (Fig. 1), which are also the areas with the highest presence of foreign residents [13, 30]. By contrast, Zone 1 (historic center) registers the lowest BMI levels among children and parents and also fewer foreign residents.

## Conclusions

In conclusion, our epidemiological research confirms the relevance of socio-cultural aspects as modifiable factors impacting diet-related diseases in a school-age children population.

Despite the low prevalence of overweight and obesity in our sample, as compared to that of the Italian national average, prevention programs for both, underweight and overweight, in primary school children should be encouraged. To implement effective interventions among children and their families, factors such as physical activity, psycho-social factors and food habits should be investigated in future research.

## References

[1] Wang Y, Lobstein T (2006). Worldwide trends in childhood over-weight and obesity. Int J Pediatr Obes.

[2] Whitaker RC, Wright JA, Pepe MS (1997). Predicting obesity in young adulthood from childhood and parental obesity. N Engl J Med.

[3] Nathan BM, Moran A (2008). Metabolic complications of obesity in childhood and adolescence: more than just diabetes. Curr Opin Endocrinol Diabetes.

[4] Bracale R, Labruna G, Finelli C (2012). The absence of polymorphisms in ADRB3, UCP1, PPARγ, and ADIPOQ genes protects morbid obese patients toward insulin resistance. J Endocrinol Invest.

[5] Deckelbaum RJ, Williams CL (2001) Childhood obesity: the health issue. Obes Res 9:239S–2343S 10.1038/oby.2001.125 11707548

[6] Dennison BA, Edmunds LS (2008). The role of TV in childhood obesity. Progress Pediatr Cardiol.

[7] Lawlor DA, Timpson NJ, Harbord RM (2008). Exploring the developmental overnutrition hypothesis using parental–offspring associations and FTO as an instrumental variable. PLoS Med.

[8] Lob-Corzilius T (2007). Overweight and obesity in childhood—a special challenge for public health. Int J Hyg Environ Health.

[9] Fredriks AM, Van Buuren S, Hira Sing RA et al (2005) Alarming prevalences of overweight and obesity for children of Turkish, Moroccan and Dutch origin in The Netherlands according to international standards. Acta Paediatr 94 (4):496–498 10.1111/j.1651-2227.2005.tb01923.x 16092466

[10] Cairella G, Casagni L, Lamberti A (2008). Prevalenza di sovrappeso ed obesità in Italia nella fascia di età 6–11 anni. Ann Ig.

[11] Albertini A, Tripodi A, Fabbri A (2008). Prevalence of obesity in 6- and 9-year-old children living in Central-North Italy. Analysis of determinants and indicators of risk of overweight. Obes Rev.

[12] Binkin N, Fontana G, Lamberti A (2010). A national survey of the prevalence of childhood overweight and obesity in Italy. Obes Rev.

[13] Comune di Milano. Popolazione residente anno 2011. Settore Statistica e S.I.T.-Servizio Statistica http://www.comunedimilano.it

[14] Stunkard AF, Albaum JM (1982) The accuracy of self-reported weights. Am J Clin Nutr 34:1593–1599 10.1093/ajcn/34.8.1593 7270483

[15] Cole TJ, Bellizzi MC, Flegal KM (2000). Establishing a standard definition for child overweight and obesity worldwide: international survey. BMJ.

[16] Cole TJ, Flegal KM, Nicholls D (2007). Body mass index cutoffs to define thinness in children and adolescents: international survey. BMJ.

[17] Spinelli A, Baglio G, Cattaneo C et al Gruppo OKkio alla SALUTE; Coorte PROFEA anno 2006 (2008) Promotion of healthy lifestyle and growth in primary school children (OKkio alla SALUTE). Ann Ig 20:337–344 19014105

[18] Nyholm M, Gullberg B, Merlo J (2007). The validity of obesity based on self-reported weight and height: implications for population studies. Obesity.

[19] Sistema statistico Nazionale-Istituto nazionale di Statistica. La vita quotidiana nel 2009. Indagine multiscopo annuale sulle famiglie “Aspetti della vita quotidiana”. Anno 2009. settore famiglia e società. http://www3.istat.it/datio/catalogo/2011

[20] Istituto Superiore di Sanità. OKkio alla Salute: surveillance system and physical activity in children attending primary school. Results 2008. Rapporti ISTISAN 09/24. http://www.iss.it/binary/publ/cont/0924

[21] Johnson-Taylor W, Everhardt JE (2006). Modifiable environmental and behavioural determinants of overweight among children and adolescents: report of a workshop. Obesity.

[22] Epstein LH, Roemmich JN, Robinson JL (2008). A randomized trial of the effects of reducing TV viewing and computer use on body mass index in young children. Arch Pediatr Adolesc Med.

[23] Manios Y, Kondaki K, Kourlaba G (2009). TV viewing and food habits in toddlers and preschoolers in Greece: the GENESIS study. Eur J Pediatr.

[24] Van Strien T, Koenders PG (2012) How do life style factors relate to general health and overweight? Appetite 58:265–270 10.1016/j.appet.2011.10.001 22027272

[25] Kirchengast S, Schober E (2006). To be an immigrant: a risk factor for developing overweight and obesity during childhood and adolescence. J Biosoc Sci.

[26] Hassapidou M, Papadopoulou SK, Frossinis A (2009). Sociodemographic, ethnic and dietary factors associated with childhood obesity in Thessaloniki, Northern Greece. Hormones.

[27] Cherkaoui I, Mouane N, Ettair S (2011) Prevalence of obesity and overweight in children: a study in government primary schools in the city of Rabat, Morocco. Arch Med Res 42:703–708 10.1016/j.arcmed.2011.12.004 22227044

[28] Popkin BM, Udry JR (1998). Adolescent obesity increases significantly in second and third generation U.S. immigrants: the National Longitudinal Study of Adolescent Health. J Nutr.

[29] Hood MY, Moore LL, Sundarajan-Ramamurti A (2000). Parental eating attitudes and the development of obesity in children. The Framingham Children’s Study. Int J Obes Relat Metab Disord.

[30] Magnusson MB, Hulthén L, Kjellgren KI (2005). Obesity, dietary pattern and physical activity among children in a suburb with a high proportion of immigrants. J Hum Nutr Dietet.

